# Comparative Expression Profiling of the *Chlamydia trachomatis pmp* Gene Family for Clinical and Reference Strains

**DOI:** 10.1371/journal.pone.0000878

**Published:** 2007-09-12

**Authors:** Alexandra Nunes, João P. Gomes, Sally Mead, Carlos Florindo, Helena Correia, Maria J. Borrego, Deborah Dean

**Affiliations:** 1 Center for Immunobiology and Vaccine Development, Children's Hospital Oakland Research Institute, Oakland, California, United States of America; 2 Departamento de Bacteriologia, Instituto Nacional de Saúde, Lisboa, Portugal; 3 Department of Bioengineering, University of California at Berkeley, Berkeley, California, United States of America; 4 Department of Medicine, School of Medicine, University of California at San Francisco, San Francisco, California, United States of America; Centre for DNA Fingerprinting and Diagnostics, India

## Abstract

**Background:**

*Chlamydia trachomatis*, an obligate intracellular pathogen, is a leading worldwide cause of ocular and urogenital diseases. Advances have been made in our understanding of the nine-member polymorphic membrane protein (Pmp) gene (*pmp)* family of *C. trachomatis*. However, there is only limited information on their biologic role, especially for biological variants (biovar) and clinical strains.

**Methodology/Principal Findings:**

We evaluated expression for *pmp*s throughout development for reference strains E/Bour and L_2_/434, representing different biovars, and for clinical E and L_2 _strains. Immunoreactivity of patient sera to recombinant (r)Pmps was also determined. All *pmp*s were expressed at two hours. *pmpA* had the lowest expression but was up-regulated at 12 h for all strains, indicating involvement in reticulate body development. For *pmpD*, expression peaked at 36 h. Additionally, 57.7% of sera from infected and 0% from uninfected adolescents were reactive to rPmpD (p = 0.001), suggesting a role in immunogenicity. *pmpF* had the highest expression levels for all clinical strains and L_2_/434 with differential expression of the *pmpFE* operon for the same strains. Sera were nonreactive to rPmpF despite immunoreactivity to rMOMP and rPmpD, suggesting that PmpF is not associated with humoral immune responses. *pmpFE* sequences for clinical strains were identical to those of the respective reference strains. We identified the putative *pmpFE* promoter, which was, surprisingly, 100% conserved for all strains. Analyses of ribosomal binding sites, RNase E, and hairpin structures suggested complex regulatory mechanism(s) for this >6 Kb operon.

**Conclusions/Significance:**

The dissimilar expression of the same *pmp* for different *C. trachomatis* strains may explain different strain-specific needs and phenotypic distinctions. This is further supported by the differential immunoreactivity to rPmpD and rPmpF of sera from patients infected with different strains. Furthermore, clinical E strains did not correlate with the E reference strain at the gene expression level, reinforcing the need for expansive studies of clinical strains.

## Introduction

*Chlamydia trachomatis* is an obligate intracellular pathogen that is responsible for significant worldwide morbidity associated with ocular and sexually transmitted diseases (STD). The developmental cycle of the organism is biphasic beginning with the adhesion of the elementary body (EB), an infectious and metabolically inert form, to the host cell. After endocytosis, the EB differentiates ∼2 to 12 h post infection (p.i.) into a larger, non-infectious and metabolically active reticulate body (RB), which initiates intracellular replication by binary fission within a vacuole called an inclusion body. At ∼30 to 36 h p.i., RBs reorganize into new EBs, which are released by host cell lysis or exocytosis at 48 to 72 h p.i. that initiates another infectious cycle [1], [2].

The disease spectrum of *C. trachomatis* ranges from conjunctivitis and ocular trachoma to tubal factor infertility, ectopic pregnancy and infant pneumonitis [3], [4]. *C. trachomatis* serological variants (serovars) are grouped within two human biological variants (biovars) according to characteristics of disease presentation: the trachoma biovar, including serovars A to C and Ba, which cause conjunctivitis and trachoma, and serovars D to K and Ba, Da, Ia and Ja, which cause conjunctivitis, urogenital infections and infant pneumonitis, and the lymphogranuloma venereum (LGV) biovar (serovars L_1_ to L_3_ and L_2a_). The latter serovars are more invasive, causing genital ulceration, lymphadenitis and proctitis [3], [5]. However, serotyping of the major outer membrane protein (MOMP), and phylogenetic reconstructions of this protein and the corresponding gene (*ompA*) [6], [7] do not group serovars by trachoma, non-invasive urogenital or invasive LGV disease groups.

The molecular mechanisms behind these biological differences among serovars (or strains) are not well understood. Genome sequences of reference strains D/UW-3 [8] and A/Har-13 [9], as well as ongoing *C. trachomatis* genomic sequencing are providing information on specific genes and proteins that may explain tissue tropism and virulence differences for the three disease groups. *C. trachomatis* contains a partial tryptophan operon (*trpRBA*) where urogenital strains, but not trachoma strains, can synthesize tryptophan from mucosal substrates [10]. The toxin gene possesses an intact N-terminal region that encodes an active enzymatic domain for the urogenital strains but not for trachoma or LGV strains [11].

Research on the nine member polymorphic membrane protein (Pmp) gene (*pmp*) family has revealed phylogenetic reconstructions where six *pmp*s (*pmpB*, *pmpC*, *pmpF*, *pmpG*, *pmpH* and *pmpI*) form clades that correspond to the three disease groups [12]–[14]. At the gene expression level, previous RT-PCR analyses of reference strains D/UW-3 and L_2_/434 [15], and microarray analysis of D/UW-3 [16] found that all nine *pmp*s were transcribed starting at 8 h p.i. Yet, based on real-time quantitative (k)RT-PCR, we found expression as early as 2 h p.i. for *pmpC* for reference strains Ba/Apache-2, G/UW-57 and L_2_/434, and a differential expression profile with earlier up-regulation of *pmpC* for L_2_/434 [17]. Also, another study based on kRT-PCR, Kiselev *et al.* [18] detected *pmpD* expression as early as 2h p.i. for L_2_/434. Proteomics analyses have also shown that all Pmps of L_2_/434 are detected as outer membrane constituents [19]–[22]. There is also evidence that some Pmps are antigenic for human sera [15], [23]. We observed a heterogeneous immunoreactivity to recombinant (r)PmpC using sera from patients infected with different *C. trachomatis* strains, suggesting a role for PmpC in antigenic variation [17]. More recently, Pmps have been considered autotransporters based on bioinformatics analyses [24], [25]. Wehrl *et al*. [26] has experimentally demonstrated the autotransporter model for the *C. pneumoniae* ortholog of *C. trachomatis* PmpD, Pmp21. Further, using immunofluorescence microscopy, Western blotting and penicillin treatment, the results of Kiselev *et al.* [18] for L_2_/434 PmpD are in general agreement with the autotransporter model for this protein. PmpD has also been shown to be a species-common neutralizing antigen [27], while PmpF has been implicated as a potential target of the host immune response as it contains several predicted major histocompatibility (MHC) epitopes within the N-terminal domain [9].

Despite the potential importance of Pmps in chlamydial biology, there is a lack of expression data for the *pmp* genes as well as an insufficient understanding of the host immune response to their proteins. Here, we profile the expression of all *pmp*s throughout development for reference strains E/Bour and L_2_/434, representing the two *C. trachomatis* biovars. We chose E/Bour because it is the most prevalent strain worldwide, although the mechanisms of its ecological success are not yet understood. L_2_/434 was selected as it has been widely studied with a plethora of biological information for comparative analyses. The biological uniqueness of these two strains *in vivo* is reflected in their differential tissue tropism, virulence and disease presentation. In light of our recent findings that reference strains do not represent the same genetic composition of clinical strains that are circulating among human populations today [28], we also compared the nine *pmp* expression levels for four C. *trachomatis* clinical strains, representing *ompA* genotypes of E and L_2_. Further, we examined the immunoreactivity of sera from adolescents with and without *C. trachomatis* urogenital infections against rPmps to further define their potential importance in human disease.

## Materials and Methods

### *C. trachomatis* cell culture of reference strains and clinical strains

*C. trachomatis* reference strains E/Bour and L_2_/434, three clinical strains belonging to *ompA* genotype E (designated as E/537C-05, E/S-141 and E/CS-500-96) and one clinical strain belonging to *ompA* genotype L_2 _were evaluated in this study. E/537C-05 and E/S-141 were collected from patients with vaginal discharge, E/CS-500-96 from a patient with cervicitis, and L_2_ from a patient with proctitis. Each was propagated in HeLa 229 cell monolayers using standard techniques as previously described [4], [29]. EBs were harvested at 48–72 h p.i. and purified by discontinuous density centrifugation in Renografin [30].

Confluent HeLa cells were either mock-infected or infected with a multiplicity of infection of one for each reference strain or clinical strain in SPG prior to incubation with culture medium [4], [29]. Eight T_25_ flasks (one for each time point of 2, 6, 12, 18, 24, 36, and 48 h and mock-infected) per strain were inoculated and placed at 37°C in 5% CO_2_ [17]. Cultured cells were harvested at each time point, and total RNA was extracted as previously described [17].

### Reverse Transcription and Quantitative Real-Time PCR (kPCR)

RNA was quantified by O.D. measured at A_260_. cDNA was generated from 500 ng of each RNA sample using TaqMan RT Reagents (Applied Biosystems, Foster City, CA) and random hexamers, and was quantified by O.D. measured at A_260_.

Quantitation of *pmp* expression was achieved using the ABI 7000 SDS (Applied Biosystems), SYBR Green chemistry, and the standard curve method for relative quantitation, using reagents and thermocycling as previously described [17]. *16SrRNA* was used as the endogenous control since normalizing the data against *16SrRNA* provides a control for the number of organisms (EBs and RBs) and, therefore, for the differential growth rate of each strain. *ompA* was included as a quality control for kRT-PCR results since it has been widely used for gene expression studies [16], [17].

Primers for each of the nine *pmp*s (Table 1) were designed using Primer Express (Applied Biosystems). Primers for *ompA*, *16SrRNA*, and *pmpC* were used as previously designed (Table 1) [17].

**Table 1:** Oligonucleotide primers used for kRT-PCR

Gene	Primers	Primer sequence (5′ to 3′)	Gene Location	Base pair size
*pmpA*	pmpA-3 a	TGCTAGGGAAGATGTTGCAAATAG	1434–1457	51
	pmpA-4 a	TGAACGGGTTGGTTAAAAATCG	1484–1463	
*pmpB*	pmpB-5 a	CGACTATCAGCAAAAACACTGCTAA	2120–2144	102
	pmpB-6 a	TAGCGGAGTTCTCAGAGATATTCAGTT	2221–2195	
*pmpC*	pmpC-11 a	TTAGTGCTCCCTACAGACTCATCAA	4150–4174	56
	pmpC-12 a	CCCGTCAGTACTATTTTCTGAGCTT	4205–4181	
*pmpD*	pmpD-3 a	GCGTGTCGCTCTGGAAAATAAT	4455–4476	51
	pmpD-4 a	ACTGTGCTGAAGTAAGAACTCCATTC	4505–4480	
*pmpE*	pmpE-1 a	CATATGCGCTCTTCCGGATAC	2140–2160	51
	pmpE-2 a	GTGTGTCTGCCCTGCTATCATC	2190–2169	
*pmpF*	pmpF-5 a	TCCTATGTTTGATCGCATTGCT	2520–2541	69
	pmpF-6 a	CTCCGCATGTTATGTGTTCCA	2588–2566	
*pmpG*	pmpG-1 a , b	TGGGTTTCTGGAGTTTCGAATT	2221–2242	51
	pmpG-2 a , b	ACCTAAAGCATCGCGGTCAT	2271–2252	
	pmpG-3 a	TGTGGCCCTGTACAATTCTTAGG	1165–1187	52
	pmpG-4 a	AAATCGCTCCACCATCATTAGC	1216–1195	
*pmpH*	pmpH-15 a	TGCATACGCAGTATTTTAATGACAAA	2486–2511	61
	pmpH-16 a	TGCCAATGACATTTCGAATGAT	2546–2525	
*pmpI*	pmpI-1 a	GGAGAAGTGTGCGCATCGAT	2176–2195	51
	pmpI-2 a	GAACAGTCCGGAACCATTGG	2226–2207	
*ompA*	OmpA-9 c	TGCCGCTTTGAGTTCTGCTT	33–52	75
	OmpA-10 c	GTCGATCATAAGGCTTGGTTCAG	108–86	
*16SrRNA*	16SRNA-9 d	GCGAAGGCGCTTTTCTAATTTAT	734–756	76
	16SRNA-10 d	CCAGGGTATCTAATCCTGTTTGCT	809–786	

a Primers designed based on each *pmp* sequence of reference strains E/Bour and L_2_/434 [12].

b Primers designed only for strain L_2_.

c Primers designed based on the *ompA* sequence of reference strains E/Bour and L_2_/434 (GenBank Accession No. X52557 and M14738, respectively).

d Primers designed based on the *16SrRNA* sequence of reference strains E/Bour and L_2_/434 (GenBank Accession No. D85722 and U68443, respectively).

Each plate contained two replicates of each sample cDNA, three different negative controls and standard curves for each gene as previously described (17). For all experiments, the amount of target and control gene was determined from the respective standard curve by conversion of the mean threshold cycle values. Normalization was obtained by dividing the quantity of the target gene by the quantity of the control gene. The specificity of the amplified products was verified by analysis of the dissociation curves generated by the ABI7000 software based on the specific melting temperature for each amplicon. The results were based on three independent experiments for reference strains E/Bour and L_2_/434, and for the four clinical strains.

### Genetic analysis of the *pmpFE* operon for *C. trachomatis* reference and clinical strains

Based on the considerable expression disparities between *pmpF* and *pmpE* (which belong to the same operon) for reference strain L_2_/434 and mostly for the clinical strains (see results below), we sequenced the *pmpFE* operon as well as the upstream 164 base pair (bp) *pmpG/pmpF* intergenomic region (IGR) that likely contains the operon regulatory region. In the *C. trachomatis* chromosome, *pmpF* and *pmpE* are located on the minus strand; *pmpF* is located upstream of *pmpE*, with a 2 bp IGR . *pmpE* was sequenced for the six strains (Genbank Accession Numbers EF490370 for E/537C-05, EF490371 for E/CS-500-96, EF490372 for E/S-141, EF490373 for L2, EF490374 for E/Bour, and EF490375 for L_2_/434), while *pmpG/pmpF* IGR and *pmpF* were sequenced only for the clinical strains (Genbank Accession Numbers EF490366 for L2, EF490367 for E/CS-500-96, EF490368 for E/S-141, and EF490369 for E/537C-05), as the sequences for the reference strains were available from our previous study (GenBank Accession Number AY887650 for E/Bour and AY887660 for L_2_/434) [12]. The amplification and sequencing strategies were performed as previously described [12], except for the *pmpG/pmpF* IGR, where we used primer 5′-ACT CGG ATC TCC TAT AAC AG-3′ for sequencing.

Since the transcription process can be strongly affected by the structure and sequence variability of promoter regions [31]–[34], a putative promoter search for the *pmpFE* operon was performed using EditSeq software (DNASTAR, Madison, WI) for sequences described in the literature and also by using two promoter prediction programs: http://www.fruitfly.org/seq_tools/promoter.html and http://www.prodoric.de/vfp/vfp_promoter.php. Given the expression dissimilarities obtained in this study for *pmpF* and *pmpE*, we searched for putative Shine-Dalgarno ribosome binding sequences (RBS) [35] as well as previously described chlamydial RBS [36]–[39] within this operon, since ribosomes can either prolong or shorten the lifetime of mRNA in response to events that occur during translation or termination processes [40]. The existence of putative consensus cleavage sites for RNase E [41]–[43], the major endonuclease that generally initiates mRNA degradation in most bacteria [44], was also examined within the *pmpFE* operon. These two analyses were performed using EditSeq software (DNASTAR). Putative stem-loop structures were searched throughout the *pmpFE* operon using GeneQuest software (DNASTAR) and RNAstructure software version 4.4 (http://rna.urmc.rochester.edu/rnastructure.html) due to the regulatory or processing role of stem-loop structures in premature transcription termination as well as in mRNA degradation and maturation mechanisms [45]–[49], respectively.

### Immunoreactivity of patient sera against Pmp fusion proteins

We generated fusion proteins for PmpD and PmpF because the latter displayed such high mRNA expression for L_2_/434 and the clinical strains, and the former was expressed late in development for all strains under study, being the last up-regulated protein for four of the six strains analyzed. Also, PmpD has been associated with neutralizing epitopes [27]. The rMOMP fusion protein was available from a previous study [50]. The PET30 expression system (EMD Biosciences, San Diego, CA) was used for cloning PCR products containing *pmpD* or *pmpF* generated from strain E/Bour genomic DNA as we have described [17]. The forward and reverse primers were 5′-GACGACGACAAGATGAGTTCCGAGAAAGATATA-3′ and 5′-AATGCTGGATTGCGATTGATCTTTTAACCGGGCTTCTCCTC-3′ for *pmpD*, respectively, and 5′-GACGACGACAAGATGATTAAAAGAACTTCTCTA-3′ and 5′-AATGCAGGAGGAGCTCTGGTCTTTTAACCGGGCTTCTCCTC-3′ for *pmpF*, respectively. Sequencing confirmed that the insert was in frame with the S-tag and His-tag as we have described previously for rPmpC [17]. The clones were transformed into *E. coli* BL21 and induced using 0.1µM IPTG during the exponential growth phase. Ni-agarose (Sigma, St. Louis, MO) was used for fusion protein purification according to the package insert. Recombinant proteins were determined to be the correct molecular weight (calculated at ∼160.6 kDa for rPmpD and ∼112.3 kDa for rPmpF) by immunoblot using AP-conjugated S-protein, which binds to the S-tag peptide with a distinct band at the correct molecular weight for each. Optimal protein concentrations were determined and standardized using BCA (Pierce, Rockford, Ill) before analyzing the clinical sera. The optimal protein concentration for rPmpD was 50 ng and 100 ng for rPmpF. Sera from 39 consented female adolescents 14 to 19 years of age attending clinics in Oakland, CA, were used at a 1:50 dilution for immunoblotting as described previously [17]. The Institutional Review Board of Children's Hospital Oakland approved the study, and all patients provided written consent for all clinical samples that were obtained and used in this study. The blots were blocked with Blotto prior to reacting with patient sera and alkaline phosphatase-conjugated anti-human IgG (R&D Systems, Minneapolis, MN). The chemifluorescent substrate ECF (Amersham, Piscataway, NJ) was used to visualize reactive bands. Twenty six (67%) of the 39 adolescents were infected with a single *ompA* genotype: 3 Ba, 3 D, 8 E, 5 F, 1 G, 1 Ia, 2 J, and 3 K.

There was no evidence for mixed infections. The original cervical samples were used for sequencing (see ref 17) to best determine the presence of a mixed infection, since propagation may result in one strain overgrowing another. On inspection of the electropherograms, none of the samples had ambiguous results. All nucleotides were represented by single, clear peaks with extremely low background and without evidence for double peaks in a nucleotide position where different *ompA* genotypes differ, which might suggest a mixed infection.

## Results

### Real-time quantitation for *pmp* expression

The results of specificity assays revealed no non-specific products, and indicated the presence of the expected amplicon for each gene. Standard curves for all 11 genes had slope values between -3.1 and -3.5, which represents efficiencies between 93 and 100%. There were only minor variations in the slope for each standard curve among independent experiments, indicating a highly reproducible kPCR as we have also shown in previous experiments [17]. We defined the gene expression profile as the qualitative gene expression pattern throughout development where quantitative values are not considered. For example, one expression profile would show increasing expression up to a peak with tapering down of the expression after the peak. An expression peak was defined as the time point of the highest relative mRNA value. All quantitative expression comparisons refer to differences between the expression peak of each gene, even those occurring at different time points p.i.

### Expression profile of the nine *pmp* genes throughout development for E/Bour and L_2_/434

**Figure 1:**
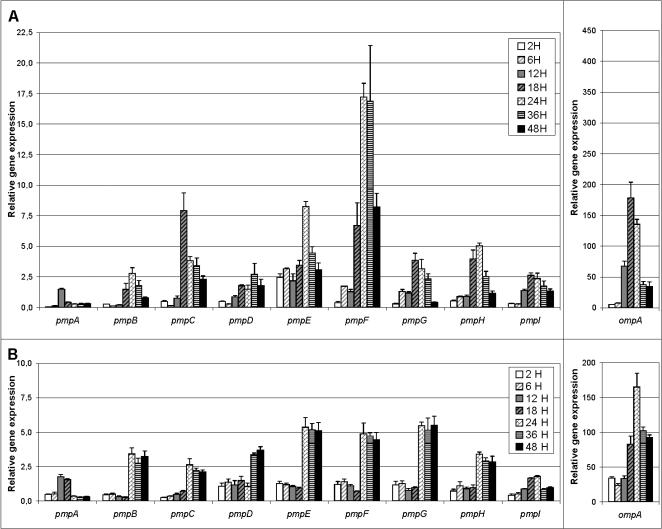
Expression profile of the nine *pmp* genes and *ompA* throughout the development of *C. trachomatis.* Reference strain L_2_/434 is represented in panel A and E/Bour in panel B. Values represent the mean±SEM based on three independent experiments for time points of 2, 6, 12, 18, 24, 36, and 48 h post infection. See methods for details.

L_2_/434 had strikingly different mRNA levels among some *pmp*s and also between different time points for the same *pmp* (Fig. 1A). *pmpF* had the highest relative mRNA expression, up to 11.5-fold higher than for *pmpA*, the least expressed gene. mRNA levels were detected at 2 h p.i. for all *pmps*, including *pmpA* where the scale limits visualization of the low mRNA expression, and peaked at different time points. For all *pmps* except *pmpA,* mRNA levels decreased consistently after the peak until 48 h.

The *pmp* expression profiles for E/Bour were more homogeneous than for L_2_/434, and, in some cases, mRNA levels were lower than for the corresponding L_2_/434 *pmp* (Fig. 1B). Similar to L_2_/434, mRNA levels were detected at 2 h p.i. for all *pmp* genes. *pmpE, pmpF* and *pmpG* showed the highest expression levels for strain E/Bour with up to 3.1-fold higher mRNA levels than for *pmpA* and *pmpI*, the least expressed genes. As for L_2_/434, *pmpA* and *pmpD* were the earliest and the latest up-regulated genes, respectively. In contrast to L_2_/434, all *pmp* genes except *pmpA* and *pmpI* had stable mRNA levels after the expression peak until 48 h. For this reason, the expression peak for E/Bour *pmps* was defined as the time point at which a noticeable expression increase occurred.

For both reference strains, *ompA* had remarkably higher mRNA values at all time points than for the *pmp*s (Fig. 1). In contrast to most *pmp*s, *ompA* revealed a similar gene expression profile for both reference strains.

### Expression profile of the nine *pmp* genes throughout development for *C. trachomatis* clinical strains

**Figure 2:**
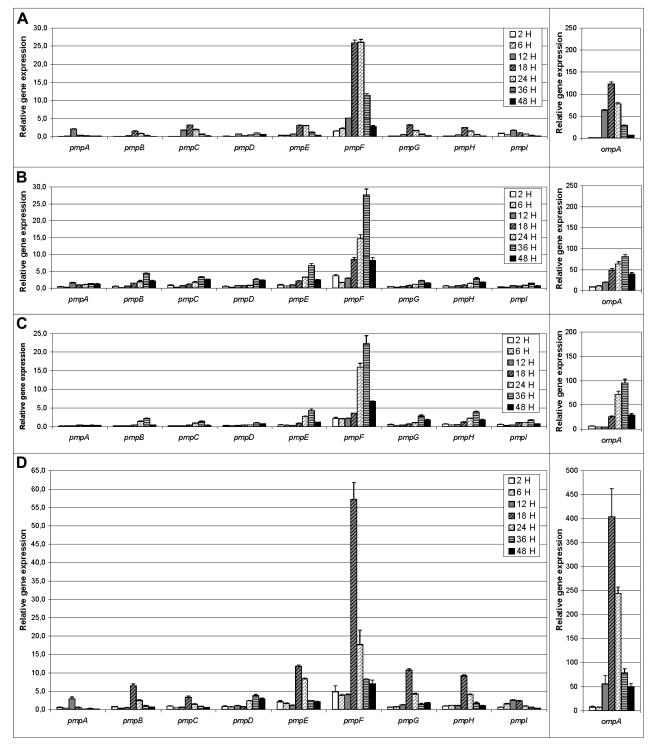
Expression profile of *pmp* and *ompA* genes throughout the development of *C. trachomatis* clinical strains. (A) Strain L_2_ shares the same *ompA* genotype as L_2_/434; and strains E/537C-05 (B), E/S-141 (C) and E/CS-500-96 (D) share the same *ompA* genotype as E/Bour. Values represent the mean±SEM based on three independent experiments for time points of 2, 6, 12, 18, 24, 36, and 48 h post infection. See methods for details.

The four clinical strains had a similar *pmp* expression profile (Fig. 2), which showed decreasing mRNA levels after the expression peak to 48 h. mRNA levels were detected at 2 h p.i. for all *pmp*s, although the scale limits visualization. Overall, *pmp*s peaked at 18h for L_2_ (Fig. 2A) and E/CS-500-96 (Fig. 2D), and at 36h for clinical strains E/537C-05 (Fig. 2B) and E/S-141 (Fig. 2C). Similar to reference strains E and L_2_, *pmpA* was the first up-regulated gene for all clinical strains. In addition, *pmpD* was the last up-regulated gene for clinical strains L_2_ and E/CS-500-96 (Fig. 2A and 2D), and was also expressed late in development (together with other *pmp*s) for the other two clinical E strains under study.

*pmpF* had the highest expression among all of the *pmps* for the clinical strains (Fig. 2). In fact, there was a 27-fold higher expression of *pmpF* compared with the least expressed gene (*pmpD*) for L_2_. For clinical E strains, there was a 19.2- and 22.6-fold higher expression of *pmpF* compared with the least expressed gene (*pmpI*) for E/537C-05 and E/CS-500-96 respectively, and a 54.2-fold higher expression than *pmpA* for E/S-141 Although no relevant dissimilarities were observed for *pmpF* between clinical L_2_ and L_2_/434, there were considerable expression differences among the clinical strains and E/Bour with up to 11.7-fold higher mRNA values for E/CS-500-96 than for E/Bour.

For *ompA*, mRNA levels peaked at 36 h for E/537C-05 and E/S-141, and at 18h for E/CS-500-96 and L_2_, declining thereafter (Fig. 2). The most striking example of differential mRNA levels between *ompA* and *pmp*s occurred for E/S-141, where *ompA* had a 232.0-fold higher value compared with the least expressed gene, *pmpA*. However, all clinical strains except E/CS-500-96 had lower *ompA* expression levels for all time points compared with the corresponding reference strains.

### Genetic analysis of *pmpFE* operon for *C. trachomatis* reference strains and clinical strains

**Figure 3:**
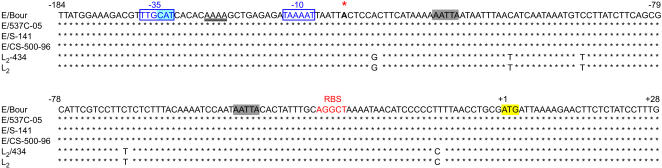
Predicted *pmpF* promoter sequence for reference and clinical strains. Sequences are for reference strains E/Bour and L_2_/434, and clinical strains E/537C-05, E/S-141, E/CS-500-96, and L_2_. The predicted transcription promoter for *pmpF* is located within a 100% conserved region of the *pmpG/pmpF* IGR, where putative -10 and -35 elements are in blue characters and boxed. Potential A/T spacer region is underlined, and the predicted transcription start site is shown in a larger font below a red asterisk. The putative RBS for *pmpF* is in orange characters, and the putative RNase E cleavage sites are highlighted in grey. Numbers represent positions relative to the start codon of *pmpF* (highlighted in yellow). The start codon of *pmpG* is highlighted in blue.

The *pmpF*, *pmpE* and *pmpG/pmpF* IGR sequences for the three clinical E strains were 100% similar to the corresponding E/Bour sequences, while L_2_ showed 4 nucleotide (nt) differences to L_2_/434 but only for *pmpE*. Compared to both L_2_ strains, the four E strains showed 317 (10.2%) nt and 106 (10.3%) amino acid (aa) differences for *pmpF* as well as 56 (1.9%) nt and 21 (20 to L_2_) (2.1%) aa differences for *pmpE*. For the *pmpG/pmpF* IGR, which comprises the ∼164 bp upstream region of *pmpF*, there were 5 nt differences between the L_2_ and the E strains, although none of them fell within the putative promoter region for the *pmpFE* operon (Fig. 3). The putative promoter is located within a 100% conserved stretch of the *pmpG/pmpF* IGR for both reference and all clinical strains (Fig. 3). The -10 promoter element (TAAAAT) identified in this study was identical to the one that was previously characterized for the L_2_/434 and D/UW-3 *ltuB* promoter, while the -35 region (TTGCAT) was 100% similar to the *hctA* promoter of the same chlamydial reference strains [32].

**Figure 4:**
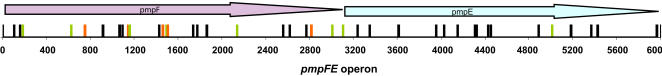
Distribution/Location of the putative RNase E cleavage sites within the *pmpFE* operon coding sequence. The sequence is for reference strains E/Bour and L_2_/434 and for clinical strains E/537C-05, E/S-141, E/CS-500-96 and L_2_. Black vertical lines represent all RNase E cleavage sites conserved among all strains under study; green vertical lines show the ones only conserved among the four “E” strains; orange vertical lines represent those specific solely for both L_2_ strains. Numbers represent nucleotide positions relative to the start codon of *pmpF.*

Analysis of the *pmpFE* operon sequence revealed several putative hairpin loop structures although the actual RNA folding in those regions functioning as a classic rho-independent type transcriptional terminator [49] cannot be assumed. At least 41 putative RNase E cleavage sites were identified throughout the *pmpFE* operon, 13 of which were not conserved between L_2_/434 (and L_2_) and the four E strains (Fig. 4). One of these non-conserved sites involved the *pmpF/pmpE* IGR, and is specific for the E strains. The search for an RBS revealed a perfect prokaryotic Shine-Dalgarno sequence (AGGAGG) located 17 nts upstream of the start codon of *pmpE,* which is approximately 3000 bp below the last bp in Figure 3 and, therefore, is not shown. This RBS is in close proximity to the above-described putative RNase cleavage site shared only by the four E strains. However, the best approach for a putative RBS sequence for *pmpF* has two mismatches when compared with the ones described in the literature, and is unusually distant from the start codon (Fig. 3). Two additional putative RNase E cleavage sites, one of which was in close proximity to this RBS, were identified within the *pmpF* regulatory region (Fig. 3).

### Immunoreactivity of patient sera with Pmp fusion proteins

**Figure 5:**
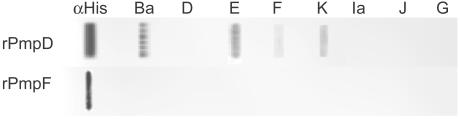
Dot-Blot of serum immunoreactivity against recombinant (r)PmpD and rPmpF. Sera was obtained from adolescents singly infected and uninfected with a different *C. trachomatis* clinical strain as described previously [17] (see also methods). rPmpD and rPmpF concentrations were standardized for use on the blots. Immunoreactivity to each fusion protein for sera from patients infected with strain Ba (n = 3), D (n = 3), E (n = 8), F (n = 5), G (n = 1), Ia (n = 1), J (n = 2) or K (n = 3) are shown. Of note is that immunoreactivity was consistent for sera from patients infected with the same clinical strain except for strain F (Table 2); all eight patients infected with strain E were reactive to rPmpD.

Table 2 shows the clinical and microbiologic characteristics of the 39 adolescents enrolled in the study and the results of their serum immunoreactivity to rPmpD and rPmpF. All sera from patients infected with chlamydial clinical strains Ba, E, F and K (n = 15; 57.7%), but none with D, Ia, J or G (n = 11; 42.3%), were reactive to rPmpD while sera from uninfected patients were nonreactive with rPmpD (p = 0.001). Figure 5 shows the immunoblot results of representative sera from patients infected with Ba, D, E, F, G, Ia, J and K to rPmpD. Because *pmpD* is highly conserved among all reference strains [12], constructing rPmpD using the *pmpD* sequence of reference strain E/Bour should not have contributed to the observed differences in immunoreactivity. Further, cross-reactivity between strains was unlikely since the patients were infected with only a single strain, and sera that were reactive to rPmpD were not reactive to rPmpF. In our previous study, sera form the same individuals infected with clinical strains D, E and G reacted with rPmpC [17]. Surprisingly, none of the sera reacted to rPmpF (Figure 5), not even sera from the eight patients infected with strain E, although all sera from infected patients and one uninfected patient reacted with rMOMP as previously shown [17].

**Table 2:** Clinical and microbiologic characteristics of female adolescents from whom sera was used for determining the immunoreactivity against rPmpD and rPmpF

*ompA* genotype^a^ (n)	Clinical diagnosis^b^ (n)	Immunoreactivity of sera against recombinant fusion proteins	
		rPmpD (%)	rPmpF (%)
Ba (3)	Cervicitis	3/3 (100)	0/3 (0)
D (3)	Cervicitis Discharge^c^ (1/3)	0/3 (0)	0/3 (0)
E (8)	Cervicitis	8/8 (100)	0/8 (0)
F (5)	Cervicitis Discharge^c^ (4/5)	1/5 (20)	0/5 (0)
G (1)	Cervicitis Discharge^c^ (1/1)	0/1 (0)	0/1 (0)
Ia (1)	Cervicitis	0/1 (0)	0/1 (0)
J (2)	Cervicitis	0/1 (0)	0/1 (0)
K (3)	Cervicitis	3/3 (100)	0/3 (0)
Uninfected (13)	No clinical signs or symptoms	0/13 (0)	0/13 (0)

a Patients were adolescents 14–19 years of age who had a *C. trachomatis* infection with only one *ompA* genotype as described in methods or were uninfected;

b The diagnosis of cervicitis was based on physical findings consistent with cervicitis as determined by the examining physician; all adolescents infected with *C. trachomatis* had cervicitis, and none of these patients complained of any symptoms;

c A cervical discharge was noted by the examining physician; none of these patients had clinical signs or symptoms consistent with upper genital tract disease.

## Discussion

In this study, we determined the gene expression profile of the nine *pmp*s throughout development for reference strains L_2_/434 and E/Bour, and four clinical strains belonging to *ompA* genotypes E and L_2_. The reference strains had significant gene expression differences where E/Bour had relatively lower mRNA levels and generally sustained expression from 24 to 48 h compared with L_2_/434 (Fig. 1). Surprisingly, in contrast to clinical L_2_, the three clinical E strains showed a dissimilar *pmp* expression profile to E/Bour (Fig. 2). These remarkable expression dissimilarities are generally supported by our recent comparative genomics findings where the laboratory adapted reference strains did not reflect the same genetic make-up of strains that are circulating among human populations today and currently exposed to immune selection [28].

It is well known that the developmental stages for reference E strains occur at later time points than for reference L_2_ strains [51] **.** This is supported by the *ompA* expression for E/Bour, which is shifted ∼6 hours later than for L_2_/434. However, the differential growth rate between these two reference strains does not explain the dissimilar *pmp* expression, as most *pmp*s were up-regulated at the same time point for both (Fig. 1). For E/Bour, almost all of the *pmp*s had increased expression during the second half of development with comparable mRNA levels at these stages, suggesting a similar involvement in RB division and RB to EB transformation. For L_2_/434, although most *pmp*s showed a general up-regulation of transcription at the exponential growth phase of RB division when new membranes are being formed, *pmpC, pmpE and pmpF* appeared to play a more important role during this phase. Thus, the gene expression results of both E/Bour and L_2_/434 suggest their potential importance in membrane integrity. However, some Pmps may have more specific functions than others, depending on the chlamydial strain. In support of this, a proteomics study by Shaw *et al.* [21] detected five Pmps among reference strains A/HAR-13, D/UW-3, and L_2_/434, where PmpF was only detected for L_2_/434. In another proteomics study, only *pmpE*, *pmpG*, and *pmpH* were detected for L_2_/434 [22]. However, it is possible that these studies reflect a lack of sensitivity in detecting Pmps since a recent study was able to detect all Pmps for L_2_/434 [19].

Interestingly, *pmpA* had, in general, the lowest expression levels of all *pmp*s at each time point except that it had one of the highest levels at 12 h p.i. (Fig. 1 and 2), suggesting a greater importance of PmpA during early stages of development. This is supported by shotgun proteomics where Skipp *et al.* [19] identified PmpA exclusively in RBs, whereas all other Pmps were detected in both RBs and EBs for L_2_/434. Additionally, for PmpD, the late up-regulation at 36 h corresponds to RB transformation into EBs, suggesting a role in EB outer membrane structure. In support of this, PmpD has a cysteine content considerably higher than any other Pmp [12]. There are 26 conserved cysteine residues in PmpD for all 19 *C. trachomatis* reference strains, while the mean for all other Pmps is only 13.9 [SE 2.3]. Cysteine residues are responsible for the highly disulfide cross-linked proteins of the outer membrane complex of EBs. Previous studies found that PmpD is surface located and cross-linked in the chlamydial outer membrane complex through disulfide bonds [52]. Furthermore, the N-terminal domain of *C. pneumoniae* Pmp21, the *C. trachomatis* PmpD ortholog, was shown to be non-covalently bound to other components of the EB surface [26]. Additionally, PmpD has shown species-specific neutralizing activity [27]. These collective data are supported by our findings that sera from *C. trachomatis* infected patients were reactive to rPmpD (Fig. 5). Our results were remarkably consistent for sera from patients infected with the same strain. For example, sera from all eight patients infected with strain E were reactive as were sera from three patients infected with strain Ba and three infected with strain K, although only one of the five patients with strain F were reactive; none of patients with strains D, Ia, J or G were reactive. Additional research is required to determine epitopes on PmpD that may correlate with the differential immune responses we observed.

Overall, considering both reference strains and clinical strains, *pmpA* and *pmpI* were the least expressed genes, while *pmpF* was the most highly expressed, although *pmpE* and *pmpG* also had similar expression levels for E/Bour. We previously found that PmpF is the most polymorphic protein among the *C. trachomatis* Pmps for both reference and clinical strains [28], [12]. Consistent with the observed protein diversity, phylogenetic analyses of PmpF grouped *C. trachomatis* strains by tissue tropism properties [12]. Further, comparative analyses of PmpF reveal distinct domains that may be associated with a specific disease group.

The outer membrane exposure of the N-terminus has been experimentally demonstrated for some *C. pneumoniae* Pmps [26], [53], suggesting that these proteins may be subjected to host immune pressure. The N-terminal half for *C. trachomatis* PmpF also contains numerous non-synonymous amino acid changes at locations of predicted MHC epitopes [9], indicating that it may be involved in eliciting a cellular immune response. Our findings that none of the sera from infected patients reacted with rPmpF suggest that this protein is not associated with the humeral immune response. Strain origin (E/Bour) of rPmpF did not seem to be an issue as sera from the eight patients infected with strain E were non-reactive. Furthermore, sensitivity was unlikely to be an issue given the immunoreactivity of the same sera with rPmpC and rMOMP, as we have previously described [17], and with rPmpD in this study. The occurrence of highly repeated GGAI motifs in the N-terminus suggests that Pmps may be associated with cell adhesion [54], which has been reported for Pmp21 of *C. pneumoniae* [26]. These cumulative findings suggest that Pmps are expressed with a differential immune response for patients infected with a specific strain. These findings and the remarkable *pmpF* expression dissimilarities among L_2_/434, E/Bour and the clinical strains suggest that there may be differential biological functions across strains and within the same strain for PmpF, either as a structural component to maintain membrane integrity, as part of a large pool of polymorphic antigens to elicit cellular immunity, or as an adhesin.

In our study, **t**he *pmpF* sequences for the three clinical E strains were found to be 100% similar to the E/Bour sequence as was L_2_ to L_2_/434. Since it is highly unlikely that identical proteins have diverse functions, we hypothesized that there may be differential regulation at the promoter level or regulation involving variations in mRNA processing and/or degradation, which would yield distinct mRNA amounts according to strain-specific needs. It is well known that point mutations in regulatory regions, such as promoter regions and RBS, can affect transcription and translation levels. However, analysis of the putative promoter region and RBS for *pmpF* showed that they are 100% conserved for both reference and the clinical strains (Fig. 3), suggesting that the observed *pmpF* expression heterogeneity may result from variations in mRNA processing and/or degradation. In fact, regulatory systems of gene expression acting at both the transcriptional and translational levels are well represented in the chlamydial genome, including homologues of endoribonucleases E, III, G and P, exoribonucleases II and PNPase, and oligoribonuclease [8], [9]. These are known to control mRNA stability and processing as well as translational efficiency in other bacteria, such as *Escherichia coli* and *Staphylococcus aureus* [44], [48], [55]–[57]. The susceptibility of mRNA to ribonuclease attack may be influenced by events occurring not only at any stage during ribosome binding, but also during translation elongation or termination [40].

We identified two conserved putative RNase E cleavage sites in the *pmpG/pmpF* IGR, one of which is in close proximity to the putative RBS (Fig. 3). It is known that RBS sequence variability and sequestering by competitive regulatory proteins or conformational impediments can affect ribosome binding/loading and, thus, mRNA lifetime [40]. Considering this, a hypothetical initial cleavage by RNase E could reduce the affinity of the *pmpF* translation initiation region for ribosomes, thereby allowing subsequent mRNA degradation/processing by endo- and exonucleases, preferentially for E/Bour when compared to the other strains. A similar regulation has already been reported for *sodB* mRNA of *E. coli* at low iron concentrations [58]. However, this hypothetical mechanism, although possible, is speculative and lacks experimental evidence.

*pmpF* and *pmpE* belong to the same operon, yet had remarkably dissimilar mRNA levels for L_2_/434, and more so for all clinical strains with up to 8.4-fold higher expression for *pmpF* than for *pmpE* (Fig. 1A and 2). This did not occur for the *pmpGH* operon. We speculated that the expression heterogeneity within the *pmpFE* operon may be generated by premature termination of transcription, rapid mRNA processing, or mRNA degradation primarily of the downstream gene (*pmpE*) of this large operon transcript (>6 Kb). Similar regulatory mechanisms have already been suggested to explain the existence of multiple transcripts within other bacterial policistronic operons [55], such as those of *Bacillus subtilis ara* [59], *Nitrosomonas europaea cbb* [60], and *Borrelia burgdorferi ospAB* and *bmpAB* [61], [62].

Although we cannot assume that the putative stem-loop structures found within the *pmpFE* operon sequence may act as classic rho-independent type transcriptional terminators [49], the possibility of hairpin formation (a common phenomenon in mRNA, mainly on large transcripts) cannot be ignored nor can its hypothetical processing role in mRNA degradation and maturation be discounted. Furthermore, several putative RNase E cleavage sites were identified throughout the *pmpFE* operon (Fig. 4), which is expected for policistronic operons, although it is well known that RNase E cleaves mRNA only at a limited number of sites [55]. Interestingly, some of the RNase E sites were not conserved between L_2_ and E strains, suggesting that targeted mRNA degradation or rapid processing events may occur in this large transcript. Curiously, one of these non-conserved recognition sites involved solely the *pmpF/pmpE* IGR of the four E strains. Thus, if RNase E uses this cleavage site, subsequent degradation or processing events from this point would only occur for E strains and could hypothetically yield an mRNA decay of *pmpE*. Yet, as above, this mechanism is speculative and lacks experimental evidence. However, in a previous study, differential transcript quantities were reported for the MMSO genes of *E. coli* that contained a consensus RNase E cleavage site in the intergenic regions of the operon, suggesting complex mRNA processing [63].

Overall, the heterogeneous expression levels among *pmp*s and among strains highlight the importance of this gene family in chlamydial biology. In particular, the unique expression disparity for the *pmpFE* operon with relatively high *pmpF* mRNA levels for 5 of the 6 strains under study, as well as the differential immunoreactivity of patient sera to rPmpD, suggest that some Pmps may explain phenotypic differences among strains for antigenicity, virulence and tissue tropism. Furthermore, our findings that clinical E strains do not correlate with reference strain E/Bour at the gene expression level are supported by our previously reported genomic data [28], reinforcing the need to examine clinical along with reference strains to advance our understanding of the role of *pmp*s in chlamydial biology and disease pathogenesis.
